# Vesicular Egress of Non-Enveloped Lytic Parvoviruses Depends on Gelsolin Functioning

**DOI:** 10.1371/journal.ppat.1000126

**Published:** 2008-08-15

**Authors:** Séverine Bär, Laurent Daeffler, Jean Rommelaere, Jürg P. F. Nüesch

**Affiliations:** Program “Infection and Cancer,” Abteilung F010 and Institut National de la Santé et de la Recherche Médicale U701, Deutsches Krebsforschungszentrum, Heidelberg, Germany; Yale University, United States of America

## Abstract

The autonomous parvovirus Minute Virus of Mice (MVM) induces specific changes in the cytoskeleton filaments of infected permissive cells, causing in particular the degradation of actin fibers and the generation of “actin patches.” This is attributed to a virus-induced imbalance between the polymerization factor N-WASP (Wiscott-Aldrich syndrome protein) and gelsolin, a multifunctional protein cleaving actin filaments. Here, the focus is on the involvement of gelsolin in parvovirus propagation and virus-induced actin processing. Gelsolin activity was knocked-down, and consequences thereof were determined for virus replication and egress and for actin network integrity. Though not required for virus replication or progeny particle assembly, gelsolin was found to control MVM (and related H1-PV) transport from the nucleus to the cell periphery and release into the culture medium. Gelsolin-dependent actin degradation and progeny virus release were both controlled by (NS1)/CKIIα, a recently identified complex between a cellular protein kinase and a MVM non-structural protein. Furthermore, the export of newly synthesized virions through the cytoplasm appeared to be mediated by (virus-modified) lysomal/late endosomal vesicles. By showing that MVM release, like entry, is guided by the cytoskeleton and mediated by vesicles, these results challenge the current view that egress of non-enveloped lytic viruses is a passive process.

## Author Summary

Rodent parvoviruses are non-enveloped lytic viruses that are thought excellent tools for a virotherapy of cancer because of their strong natural oncolytic potential and low pathogenicity in humans. Egress of non-enveloped lytic viruses is commonly thought to occur as a virus burst after cell disintegration. Indeed, we showed in the past that autonomous parvoviruses induce severe cytopathic effects to the host cell, manifested in restructuring and degradation of cytoskeletal filaments, thereby supporting such mode of virus spread. Here, we focus on the impact of virus-induced actin degradation, and particularly the functioning of the actin-severing protein gelsolin. Although not required for DNA replication or progeny particle production, gelsolin appears to facilitate a regulated virus egress from the nucleus to the cell periphery via (virus modified) lysosomal/late endosomal vesicles. These results challenge the current view that lytic virus egress is just a passive process at the end of infection and suggests that these pathogens are endowed with the ability to efficiently spread from cell to cell potentially in solid (tumor) tissue.

## Introduction

The genus parvovirus (PV) consists of small icosahedral non-enveloped particles with a 5.1-kb linear single-stranded DNA genome. During productive infection, PVs induce dramatic morphological and physiological changes in their host cells, culminating in cell death and lysis. PV cytotoxicity is attributed mainly to the large non-structural viral protein NS1, an 83-kDa multifunctional protein endowed with enzymatic and non-enzymatic properties enabling it to control various processes necessary for progeny particle production and spread (reviewed in [1]). To function in a concerted way, NS1 is regulated by specific phosphorylations driven mainly by members of the PKC family [2],[3]. In addition to its direct involvement in particle production, NS1 acts specifically to jeopardize the integrity and survival of infected cells [4],[5],[6]. It has been shown to control the activity and properties of selected cell components through physical interaction [7],[8] and/or induction of post-translational modifications [9],[10]. Such targets might be modified either directly by NS1/CKIIα, a recently described complex formed by NS1 with the catalytic domain of cellular CKII [8], or indirectly through activation/modulation of the PDK-1/PKC signaling cascade [11].

PV infection leads to characteristic alterations of host-cell morphology that might facilitate virus replication or the release of progeny particles. Subnuclear APAR-bodies acting as replication centers for parvoviral DNA amplification are formed early in infection [12],[13]. Later, PVs induce cytoskeletal changes evidenced by rounding-up and detachment from the culture dish prior to cytolysis [14],[15]. In MVM-infected mouse A9 cells, these morphological alterations have been attributed to the activity of NS1 [4] and shown to result from changes in micro- and intermediate filaments [10]. While tropomyosin is a direct target of NS1/CKIIα, MVM-induced actin-filament alterations appear to result from an imbalance between the polymerizing factor N-WASP (Wiscott-Aldrich syndrome protein) and gelsolin [10], a multifunctional protein known mainly for its actin-filament-severing and capping activities and its participation in processes requiring rapid actin remodeling [16]. Roles in apoptosis and lipid signaling are also reported [17]. By altering the availability of PIP_2_, gelsolin activity might interfere with PIP_2_-dependent signaling cascades affecting phospholipase C [16].

Little is known about the impact of cytoskeletal rearrangements on virus replication and spread. Cytoplasmic collapse is thought to be part of a process leading to virus release upon cytolysis [7], but there is also indirect evidence of PV release in the absence of cell disruption [18]. The aim of the present study was to assess the role of gelsolin activity and actin reorganization in PV replication and spread. We show that gelsolin-induced modulation of actin filaments is essential to virus egress and provides strong evidence that progeny virions move to the cell periphery through vesicular transport and start to be released into the medium before cell collapse at the end of infection.

## Results

### MVM-induced remodeling of gelsolin and actin filaments

**Figure 1:**
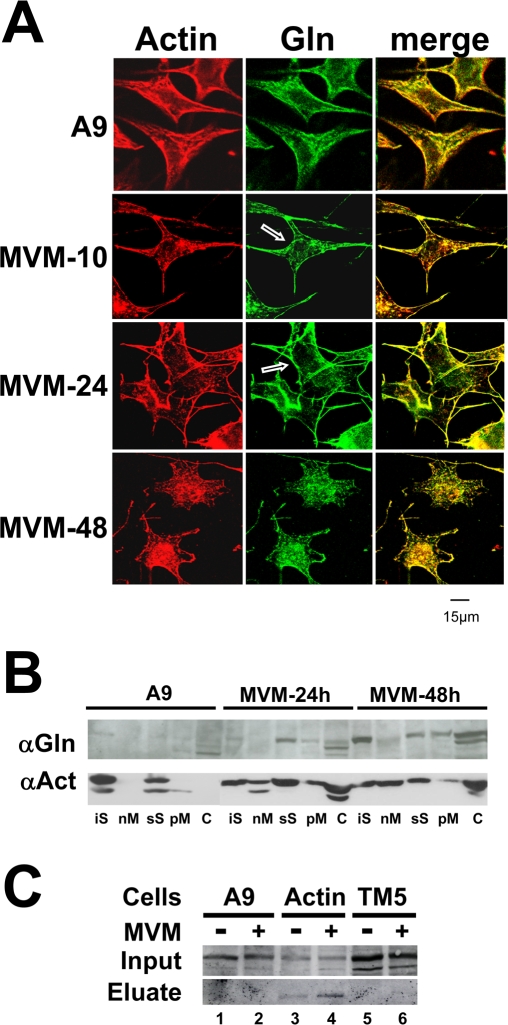
Gelsolin modulation during MVM infection of A9 cells. (A) A9 cells grown on spot slides were infected (or not) with MVM (30 pfu/cell), fixed with paraformaldehyde at the indicated time p.i., and analyzed by confocal laser scanning microscopy after double-labeling for actin with rhodamine-coupled phalloidin (red), and gelsolin, with Cy2-conjugated IgGs (green). Colocalization areas appear yellow in the merge. Scale bar 15 µm. Gelsolin is found associated to phalloidin-stained actin in both non-infected and MVM-infected cells. Arrows point to a slight but distinct accumulation of the actin-processing polypeptide in the perinuclear region. (B) A9 cells were infected with MVM (30 pfu/cell) and harvested at the indicated time p.i. Association of gelsolin and actin with cellular scaffold and membrane structures was determined by fractionating cell extracts by a combined sedimentation and Triton X-100 extraction procedure. The distribution of each protein among the various fractions was determined by western blotting. Upon MVM-infection, actin and gelsolin become associated with membrane structures. iS, insoluble scaffold proteins; nM, mainly nuclear (membrane) constituents; sS, soluble scaffold proteins; pM, mainly vesicular and plasma (membrane) constituents; C, soluble cytosolic proteins. (C) Binding of gelsolin to cytoskeletal proteins was determined by affinity chromatography using GST-tagged βactin or tropomyosin 5 (TM) as baits in stably transfected cells. Extracts of mock (−) and MVM-infected (+) cells (parental A9 [lanes 1 and 2] or derivatives expressing GST-tagged βactin [lanes 3 and 4] or TM [lanes 5 and 6]) were run through Glutathione Sepharose columns specifically retaining GST-tagged proteins and partners thereof. 700 mM NaCl eluates containing the partner proteins were analyzed by western blotting for the presence of gelsolin, in comparison with the cell-matched original extract (Input).

MVM-induced cytopathic effects include actin-fiber degradation and subsequent formation of actin patches at late stages of infection. The proposed cause is a virus-induced imbalance between actin polymerization and severing [10]. To investigate the impact of gelsolin on actin modulation and parvovirus replication we used confocal laser scanning microscopy. A9 cells infected (or not) with MVM were examined for gelsolin's subcellular distribution and its association with actin structures. Gelsolin was found to colocalize with phalloidin-stained actin structures (Figure 1A) whatever the infection status and time. In non-infected cells it accumulated abundantly along the rigid actin network and in the actin-rich region beneath the plasma membrane. Upon infection, concomitantly with destruction of the actin network, it became redistributed to the plasma membrane and perinuclear regions, later becoming associated with the above-mentioned cytoplasmic patches. The identical distribution of gelsolin and disorganized actin in infected cells suggests a link between the former and the state of the latter.

Cell fractionation experiments confirmed the above findings (Figure 1B). In non-infected cells, actin was found predominantly in the scaffold-containing fractions (iS, sS), but after infection it was found in all subcellular fractions, including the cytosol (C) and the membrane-associated fractions (nM, pM). In agreement with its association with remodeled actin structures, MVM-induced gelsolin was similarly found in all actin-positive fractions.

As additional proof of gelsolin/actin interaction during MVM-induced actin reorganization, we used affinity chromatography to study actin-gelsolin binding. This method was previously used to determine NS1 association with tropomyosin [8] and proved successful in detecting specific protein interactions with partially insoluble cytoskeleton components in the cellular context. A9 cell lines expressing GST-coupled actin under the control of the parvoviral P38 promoter were MVM or mock infected. Twenty-four hours post-infection, cell extracts were prepared, matched for the GST-actin content due to viral induction of recombinant protein expression, and passed through Glutathione Sepharose columns to trap the fusion protein and associated polypeptides. After extensive washing steps, proteins bound to the trapped GST-actin were then eluted with high salt and cell matched volumes of input and eluates were then tested by Western blotting for the presence of gelsolin. As shown in Figure 1C (actin lanes), gelsolin was invariably recovered from the GST-actin-loaded columns, whether the actin-bound proteins were from infected or non-infected cells. The specificity of the actin-gelsolin interaction was demonstrated by failure to detect gelsolin in eluates from columns loaded with extracts of A9 cells expressing only GST-free actin (A9 lanes) or GST-coupled tropomyosin (TM5 lanes). Altogether, these results strongly suggest that gelsolin can interact with both filamentous actin and virus-processed actin structures such as the cytoplasmic patches.

### Gelsolin involvement in MVM-induced actin-network remodeling and release of progeny virions

**Figure 2:**
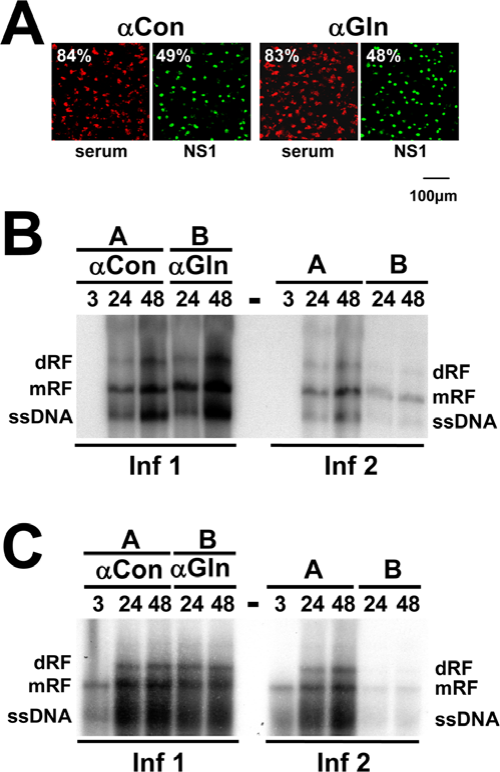
Impact of gelsolin on parvovirus replication and spreading. Cells were transfected with control serum (αCon) or neutralizing anti-gelsolin antibodies (αGln) at 7 µg/cell. Twenty-four hours post-transfection, they were infected with the indicated parvovirus, washed extensively after 2 h to remove the inoculum, and further incubated for the indicated time (h). Cells and supernatants were collected separately at the indicated time p.i. (Inf 1). To estimate the amount of infectious virions released into the medium, naive cultures were incubated with Inf-1 supernatants and harvested 24 h later (Inf 2). Although dispensible during virus entry, DNA amplification and formation of DNA-containing capsids, gelsolin appears to play a role either in the generation of infectious progeny virions and/or the release of infectious particles into the medium. (A) The efficiencies of transfection (with antibodies) and infection (with MVM) were measured in parallel by IF staining of cultured cells treated with control antiserum (αCon) or neutralizing anti-gelsolin antibodies (αGln). (B, C) Production of replicative intermediates (dRF, mRF) and single-stranded progeny-virion DNA (ssDNA) was measured by Southern blotting in (B) MVM-infected A9 cells (10 pfu/cell) and (C) H-1-PV-infected NCH149 cells (10 pfu/cell).

Since gelsolin is induced by MVM and associates with actin, we hypothesized that it might play a role in MVM-induced alteration of the actin network and in MVM propagation. To test this hypothesis, we transfected A9 cells with control serum/IgG or with antibodies (αGln) known to specifically inhibit gelsolin activity [19], infected them with MVM, and placed them in culture. At different times, cells and their medium were collected separately (Infection 1). The collected cells were tested by immunofluorescence (IF) staining for expression of the viral protein NS1, taken as indicator of successful infection (Figure 2A). Southern blots were also produced from the cells, showing the different forms of DNA typically encountered in infected cells: the double-stranded replicative forms (RF) and the single-stranded DNA (ssDNA) of progeny virions (Figure 2B, Inf1). Neither the efficiency of infection nor the levels of the different viral DNA forms appeared to be altered by the presence of gelsolin-neutralizing antibodies.

In a second phase of the experiment, the release and infectivity of progeny virions was determined by inoculating cultures of naive A9 cells with supernatant medium from infection-1 cultures (Infection 2). In this case, gelsolin activity appeared essential to either the formation or the release of infectious progeny virions, since cells exposed to infection-1 culture supernatants showed markedly lower levels of MVM DNA when the infection-1 cells had been treated with αGln (Figure 2B, Inf2). This result was confirmed by measuring production and release of infectious virions at 24 h p.i. by standard plaque assay. While cell-associated titers between αGFP-IgG and αGln-treated samples varied only marginally 1.5 fold (GFP: 2.96×10^8^, αGln: 2.12×10^8^), inactivation of endogenous gelsolin blocked release of infectious virions leading to a 30 fold reduction of medium-associated titers in αGln-treated samples (2×10^7^ vs. 6×10^5^). Similar results were obtained with human glioblastoma cells (NCH149) infected with H1-PV, indicating that dependence on gelsolin is a general, late feature of PV infection (Figure 2C).

**Figure 3:**
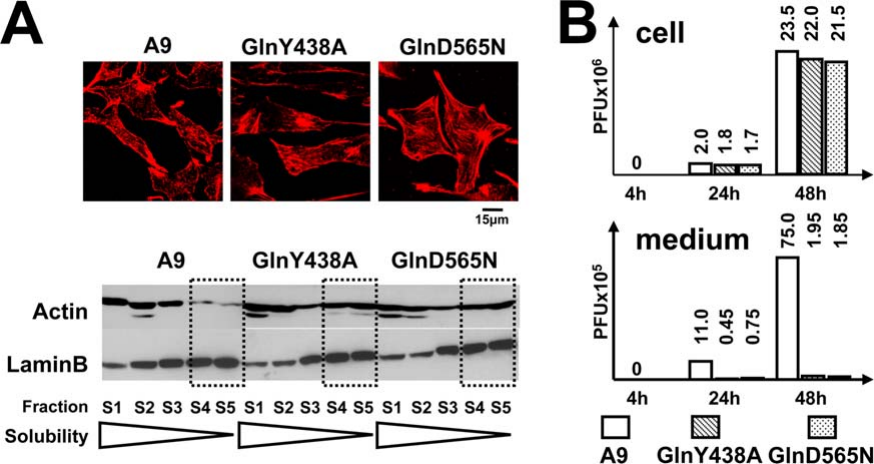
MVM production in cells expressing dominant-negative gelsolin mutants. A9 cells and derivatives expressing dominant-negative gelsolin mutants (GlnY438A; GlnD565N) were infected with MVM (30 pfu/cell). (A) Infected cells were analyzed 48 h p.i. for the structure of their actin network by IF staining with rhodamine-phalloidin (upper panel) and by biochemical fractionation according to the solubility of actin filaments (lower panel). Cell extracts were treated with detergents in increasing amount and of increasing strength, and actin was quantified in the individual fractions by western blotting. LaminB served as a loading control. Fractions containing the most rigid filaments (i.e. insoluble components) are highlighted by dotted squares. Expression of the two gelsolin variants GlnY438A or GlnD565N, respectively, protect actin filaments from degradation through parvovirus MVM, i.e. exert a dominant-negative effect. (B) Cells and supernatants were collected separately at the indicated time p.i. Titers of cell-associated (cell) and released (medium) infectious virions were determined by standard plaque-assays on A9 cells and are expressed in plaque-forming units (PFU). Inactivation of gelsolin does not affect the production of infectious progeny particles, but inhibits egress of progeny virions into the medium supernatant.

Is gelsolin required for the generation or for the release of infectious progeny virions? Does its effect on progeny virion formation or release correlate with an active involvement of gelsolin in MVM-induced remodeling of the actin network? To address these questions, we generated two cell lines, each stably transfected with a plasmid, pP38-MycGlnY438A or pP38-MycGlnD565N, driving MVM-inducible expression of a dominant-negative mutant gelsolin gene, so as to block endogenous gelsolin activity. The studied mutations were respectively: (i) a tyrosine-to-alanine substitution at position 438, disrupting a phosphorylation site for Src kinases [20] regulating the PIP_2_ interaction and actin-severing activity [21] and (ii) a glutamic acid-to-glutamine substitution at position 565, disrupting a conserved Ca^2+^-binding site regulating the activity of gelsolin through conformational alterations [22]. In IF microscopy and fractionation experiments, both mutant gelsolins (GlnY438A and GlnD565N) were found to accumulate in the perinuclear region and being associated with large, insoluble scaffold structures (Figure S1), i.e., were able to interfere with the actin-processing activity of endogenous gelsolin. Furthermore, both mutant gelsolins were found to protect actin fibers from PV-induced remodeling, notably preventing the formation of patches (Figure 3A, upper panel), and to impair the degradation of rigid actin filaments (Figure 3A, lower panel). These results both confirm the dominant-negative character of the mutations introduced and demonstrate that gelsolin is instrumental in altering the actin network after PV infection.

The same stable transfectants were then used to assess the role of gelsolin in infectious virion production and release. As in the case of cells treated with gelsolin-neutralizing antibodies (Figure 2B and 2C), transfectants expressing GlnY438A or GlnD565N retained the ability to amplify MVM DNA. In contrast, the release of infectious viruses into the medium was drastically impaired (Figure S2). This lack of “free” viruses in the corresponding culture supernatants was not attributable to efficient readsorption onto neighboring cells, since it was also observed in cultures treated with neuraminidase to prevent MVM recapture. The question was thus: does gelsolin inactivation affect the formation or the shedding of infectious progeny viruses? To address this question, we determined the infectious titers of both cell-associated and released virions (Figure 3B). Cultures of transfectants expressing a mutant gelsolin displayed similar cell-associated infectious virion titers as A9 cultures, but a drastically (20- to 40-fold) reduced titer of infectious virions shed into the medium. This strongly suggests that gelsolin is essential for efficient egress of progeny virions during MVM infection.

**Figure 4:**
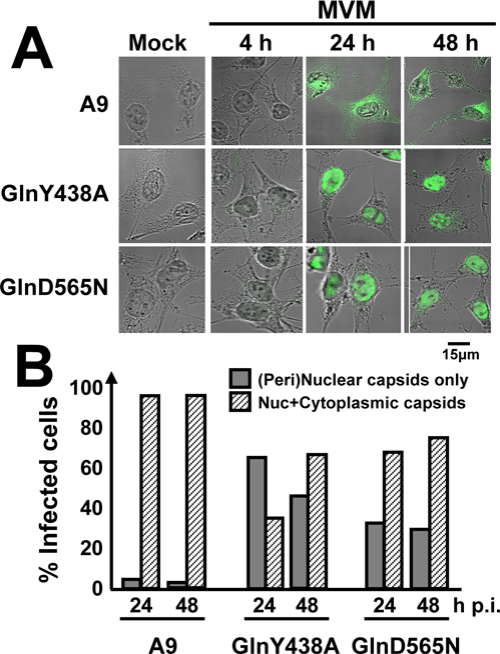
Dependence of virus egress on gelsolin. A9 cells and A9 derivatives expressing the indicated gelsolin mutants were grown on spot slides, infected (or not) with CsCl-gradient-purified MVM (30 pfu/cell), fixed at 4, 24 or 48 h p.i., and analyzed by confocal microscopy. Capsids were detected with αB7 monoclonal antibodies and cells were visualized by Nomarski staining. Inhibition of gelsolin leads to accumulation of progeny virions in the nucleus of infected cells. (A) IF staining patterns of representative cells. Scale bar 15 µm. (B) Intracellular capsid distribution, as determined by IF microscopy on at least 300 infected cells.

We next investigated the subcellular location at which progeny particles get stuck in the absence of functional gelsolin. To this end, A9 cells and cells expressing GlnY438A or GlnD565N were infected with purified MVM, fixed at the indicated time p.i., and examined by confocal microscopy for the presence of capsids. As shown for representative cells in Figure 4A and as quantified in Figure 4B, newly synthesized capsids were rapidly exported from the nucleus and transported to the periphery of A9 cells, so that capsid staining was distributed from the nucleus through the cytoplasm to the plasma membrane (Nuc+Cytoplasmic capsids). In contrast, cells expressing either of the mutant gelsolins displayed trapping of a considerable proportion of the progeny virions in or around the nucleus at least until 48 h p.i. ([Peri]nuclear capsids only).

### Pre-lytic egress of parvoviruses is mediated by vesicular transport

**Figure 5:**
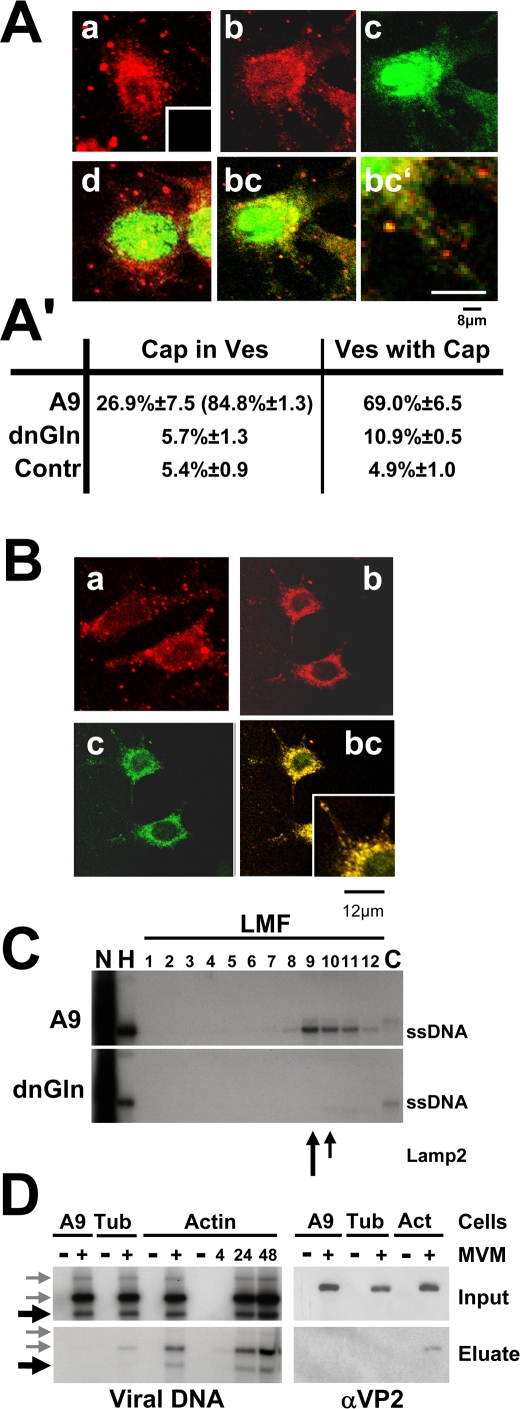
Vesicular transport of MVM progeny virions. (A, B) A9 cells and derivatives expressing a mutant gelsolin were infected (or not) with CsCl-gradient-purified MVM (30 pfu/cell), treated with neuraminidase to avoid second-round infection, and fixed at 24 h p.i. Capsids (detected with αB7 [green]) were analyzed by confocal laser scanning and spinning-disk microscopy for their subcellular localization, as compared to that of Lamp2-positive vesicles. Progeny virions are associated with cellular lysosomes and/or late endosomes as apparent from colocalization with the vesicular marker Lamp2 in the cytosol. (A) a, mock-treated A9 cells; Lamp2 (main panel) and the negative control for capsid staining (insert); b, Lamp2 staining of MVM-infected A9 cells; c, MVM capsids in infected A9 cells; bc, Lamp2/capsid merge; bc′, enlarged area; d, Lamp2/capsid merge applied to MVM-infected A9 cells expressing a dominant-negative gelsolin variant. Scale bars: 8 µm. (A′) Capsid/Lamp2 colocalization determined with imageJ, expressed as the mean value of a whole stack. Capsid/mitotracker (suppl. 3) served as a negative control (Contr.). Cap in Ves, percentage of green pixels merging with red. Values in parentheses are derived from cytoplasmic areas; Ves with Cap, red pixels merging with green. (B) a, dynamin in mock-treated A9; b, dynamin in MVM-infected A9; c, MVM capsids; bc, dynamin/capsid merge. Small squares represent enlarged areas of capsid/dynamin colocalization. Scale bars 12 µm. (C) A9 cells and cells of the derivative expressing GlnY435A were infected with MVM (30 pfu/cell), harvested at 24 h p.i., and fractionated by differential (density) centrifugation to separate different organelles. The presence of progeny particles was determined by Southern blotting (revealing their single-stranded DNA). DNA-containing progeny virions co-purify with cellular vesicles during biochemical fractionations of cellular organelles. Nuc, purified nuclei; HMF, large organelles; Cyt, cytosol. Cellular vesicles were further purified from the light mitochondrial fraction (LMF) by centrifugation through an iodixanol gradient. The migration of Lamp2 is indicated by arrows. (D) A9 cells (lanes 1&2) and derivatives expressing either GST-tagged α-tubulin (lanes 3 and 4) or β-actin (lanes 5–10) were mock-treated (−) or infected with MVM for the indicated time (h). Cell extracts were prepared and run through Glutathione Sepharose columns specifically retaining the GST-tagged proteins and their associated partners. The partners were recovered (700 mM NaCl Eluate) and tested for the presence of full (virion-containing) capsids by Southern blotting (ssDNA) and Western blotting (capsid proteins), by comparison with the corresponding total extracts (Input). Black arrows indicate the migration of ssDNA, grey arrows of free replicative form viral DNAs. DNA-containing progeny particles specifically interact with actin.

Capsid staining of infected A9 cells was noticeably spotty (Figure 4A), suggesting that progeny viruses might be transported by vesicular structures. This possibility was tested by confocal microscopy of infected cells after double IF labeling of assembled capsids and either vesicular markers or proteins known to be involved in vesicle formation. We took several measures to make sure we were observing virus release and not virus entry: (i) we checked that no incoming capsids were detected under the conditions used (Figure 4A); (ii) we prevented re-infection by neuraminidase treatment of the cells after infection; (iii) we checked that the results were similar when the cells were transfected with viral DNA rather than exposed to virus particles. In the parental A9 cells, newly synthesized capsids were found, 24 and 48 h p.i., to colocalize with Lamp2 (Figure 5A), cathepsin B, and Rab6, but not with the mitochondria (Figure S3). Cells expressing GlnY438A or GlnD565N showed no colocalization with Lamp2, cathepsin B, or Rab6. These data strongly support a role of (virus-modified) lysosomes or late endosomes in gelsolin-dependent export of progeny particles. In agreement with its involvement in (endosomal) vesicle formation [23], MVM infection caused dynamin to accumulate in the perinuclear region, where it was found to colocalize with newly synthesized capsids (Figure 5B).

To further substantiate the association of newly synthesized infectious virions with cellular vesicles, extracts prepared from infected A9 cells or A9 derivatives expressing GlnY438A were treated to separate nuclei, large organelles (HMF), a light mitochondrial fraction (LMF), and a soluble cytosolic fraction, the LMF being further fractionated in a self-forming iodixanol gradient. In A9 extracts, as shown in Figure 5C, ssDNA was found not only in the nuclei along with RF DNA, but also in the HMF and LMF, co-migrating with Lamp2, a profile suggestive of a vesicular localization. Very little virion DNA was found in the cytosolic fraction. Interestingly, only minute amounts were detected in the LMF fractions derived from cells expressing a mutant gelsolin. This suggests a possible involvement of this actin-processing protein in the formation of capsid-containing vesicles or their release from larger compartments.

To further examine the role of actin in this process we determined whether infectious virions can physically bind to (virus-modified) actin. Protein complexes formed with GST-β-actin or GST-α-tubulin were extracted from MVM- or mock-infected cells and trapped on Glutathione Sepharose columns. The partners of β-actin or α-tubulin were then recovered and the eluates tested for the presence of virion DNA (by Southern blotting) and capsid proteins (by Western blotting). Parental A9 cells served as negative controls. In contrast to free replicative form DNA which appeared to interact nonspecifically with the column material, MVM progeny virions were found to bind specifically to GST-actin and to elute from the column at high salt concentration, as evidenced by the presence of VP2 and ssDNA in the eluates from MVM-infected cells expressing GST-actin (Figure 5D). In agreement, with our findings of virus-induced actin association with (cellular) membranes (Figure 1B) and the requirement for gelsolin to egress virions from the nucleus, this supports a hypothesis that rapid actin remodeling might be required for the formation and/or motility of virion-containing vesicles.

### Regulation of gelsolin activity during MVM infection

**Figure 6:**
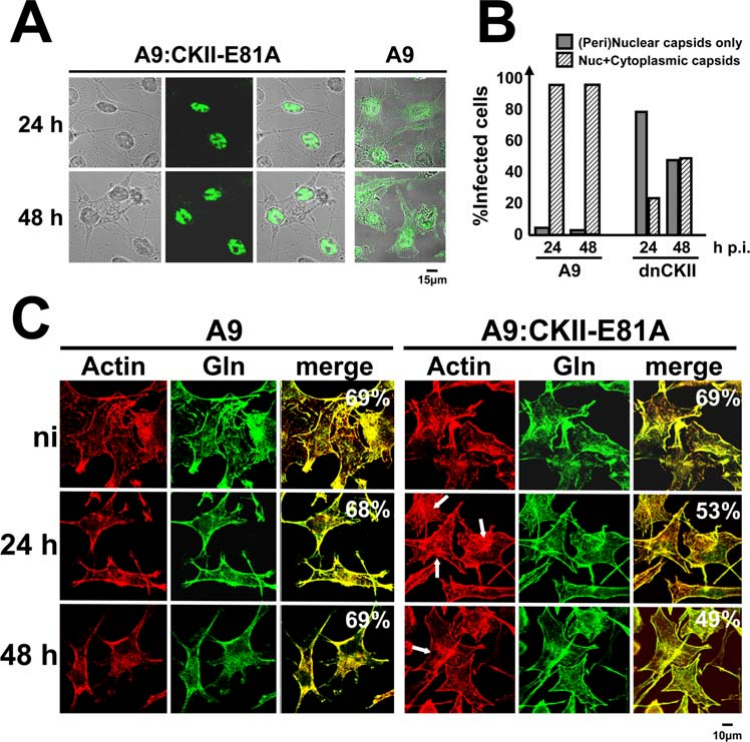
Impact of CKII on gelsolin-dependent actin processing and parvovirus egress. CKII activity is essential to promote vesicular egress of parvovirus progeny particles. A9 cells and their P38:CKII-E81A-expressing derivatives (dnCKII) were infected with CsCl-gradient-purified MVM (30 pfu/cell), fixed at the indicated time p.i., and analyzed by confocal laser scanning microscopy. (A) Capsid staining (green) within the cell (Nomarski). (B) The intracellular distribution of newly synthesized capsids was determined on at least 200 infected cells. Gray columns: percentages of cells showing a purely (peri)nuclear capsid distribution. Hatched columns: percentages of cells showing both a (peri)nuclear and a cytoplasmic capsid distribution. (C) Actin filaments stained with rhodamine-phalloidin (red) and gelsolin (green). Colocalization areas appear yellow in the merge and were quantified using ImageJ. White arrows indicate large actin fibers. CKII-activity is involved in parvovirus-induced actin processing. Scale bars: 15 µm (A) 10 µm (C).

Previous investigations with an MVM-inducible cell line expressing a dominant-negative mutant of CKII (A9-P38:CKII-E81A) have shown that functional CKII is essential to the release of progeny virions into the culture medium [7]. As illustrated in Figure 6A and quantified in Figure 6B, these cells are distinguishable from the parental line A9 by a striking retention of progeny viruses in the nucleus and perinuclear region. This defect, similar to that observed after functional inactivation of gelsolin, suggests that CKII might take part in regulating gelsolin in MVM-infected cells. This hypothesis was first tested by determining whether expression of the dominant-negative form of CKIIα can interfere with gelsolin-dependent remodeling of actin filaments in infected cells. A9 and A9-P38:CKII-E81A cells were infected with purified MVM and examined by confocal microscopy. As shown in Figure 6C, inhibition of CKIIα was found to correlate with prolonged persistence of rigid actin filaments and delayed formation of actin patches. Furthermore, gelsolin/actin colocalization was strongly reduced upon MVM infection. All of these observations are in agreement with the involvement of this kinase in controlling gelsolin-driven cytoskeletal changes.

**Figure 7:**
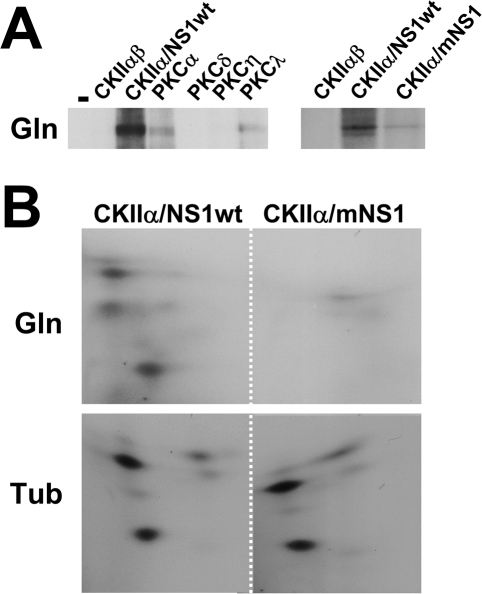
Sensitivity of gelsolin to *in vitro* phosphorylation by cellular protein kinases. Gelsolin is subject to phosphorylation through NS1/CKIIα at least *in vitro*. (A) Bacterially expressed gelsolin (Gln) was used as a substrate for the various indicated recombinant protein kinases in the presence of [^32^P]-γ-ATP. CKIIαβ was tested alone or in combination with wild-type (wt) or mutant (m:S473A) GST-tagged NS1 protein. [^32^P]-labeled gelsolin was immunoprecipitated and analyzed by 10% SDS-PAGE and autoradiography. (B) Gelsolin (Gln) and tubulin (Tub) were subjected to *in vitro* phosphorylation by CKII/NS1wt or CKII/mNS1, purified, and processed for 2-dimensional tryptic phosphopeptide analysis.

We then investigated whether gelsolin might be a target of phosphorylation by cellular protein kinases. Bacterially expressed purified gelsolin was incubated with recombinant PKC (isoform α, δ, η, or λ) or CKIIαβ in the presence of [^32^P]-γ-ATP. CKIIαβ was tested either alone or with the GST-NS1 polypeptide, given our recent finding that NS1 can act as an adaptor and modulate the substrate specificity of this kinase [8]. [^32^P]-labeled proteins were then analyzed by SDS-PAGE and autoradiography. As shown in Figure 7A, gelsolin proved to be a poor substrate for most of the tested protein kinases, including CKIIαβ alone and the NS1-modifying PKCη and PKCλ. In contrast, it was readily phosphorylated by the NS1/CKIIα complex. Two-dimensional phosphopeptide analyses confirmed that NS1 endows CKII with the capacity to phosphorylate gelsolin at multiple sites (Figure 7B). This NS1 dependence is specific, since the viral product failed to modulate the CKII-driven modification of tubulin. These results raise the intriguing possibility that MVM-induced modification of gelsolin by the NS1/CKIIα complex results in actin network alterations that facilitate virus egress.

## Discussion

Previous studies have shown that cytoplasmic collapse at the end of parvovirus MVM infection does not reflect mere cell exhaustion, but results from a readily observable remodeling of cytoskeleton filaments [10]. In contrast to tropomyosin, which is degraded upon NS1/CKIIα phosphorylation [8], and to microtubules, which are actively protected through PKCλ-mediated phosphorylation [10], actin appears to undergo remodeling as a result of a virus-induced imbalance between the activator protein for polymerization, N-WASP, and the severing factor gelsolin. We show here that gelsolin activity is required not only to trim actin filaments but also to drive virus export from the nucleus (or its immediate periphery) to the outside of infected cells. This transport of progeny virions to the cell periphery is mediated by vesicles bearing protein markers of lysosomes and/or late endosomes, suggesting that gelsolin may play a role in the formation, loading, and/or trafficking of these vesicles.

Little is known about the egress of non-enveloped lytic viruses from infected cells, commonly thought to occur as a virus burst after cell disintegration. Although cell lysis can be expected to contribute considerably to virus spread in tissue cultures, its importance in animal infection is unclear, as there is some evidence of non-lytic egress of non-enveloped viruses [24], including PVs [18]. Here we present evidence suggesting that PV egress from infected cells is controlled by a cytoskeleton-regulating protein, gelsolin, and mediated by vesicles, arguing strongly for an active egress mechanism. This process bears some resemblance to parvovirus entry, involving clathrin-dependent endocytosis and endosomal transport via microtubules [25]. For virus egress, gelsolin is essential at an early step taking place at or near the nuclear envelope. Although the transfer of incoming PVs to the nucleus is partly understood [26],[27], the gelsolin-dependent event enabling progeny virions to leave the nuclear area is currently a matter of speculation. We show here that in MVM-infected cells, gelsolin accumulates not only along rigid actin fibers but also at the plasma membrane and, at a later stage of infection, within actin patches. These findings are in agreement with the actin-processing function of gelsolin, but provide no obvious clue to its involvement in the peripheral transport of PV particles. Interestingly, rapid actin remodeling, a known gelsolin activity, is reported to be associated with the formation of vesicles [23],[28]. Our present finding that progeny virions are detectable in vesicular structures only if gelsolin is functional leads us to suggest that gelsolin may drive the assembly, loading, or mobilization of vesicles involved in transferring viral particles from the perinuclear region to the cell periphery.

There are multiple reports demonstrating actin-dependent transport of intracellular pathogens (including viruses) and cellular vesicles [24],[28]. While entry and egress of vaccinia virus involves movement along the microtubules, propulsion of this virus during dissemination from infected cells to adjacent tissue requires rapid actin polymerization induced by a WASP-like viral protein constitutively activating the Arp2/3 complex and leading to the appearance of “actin-tails” [29]. The movement of vesicles, on the other hand, has been shown to be driven by myosins along intact actin filaments [30]. Our data do not support such a role of the actin scaffold in guiding (vesicle-contained) MVM towards the plasma membrane, since (i) there is no homology between PV-encoded proteins and ENA/WASP-family proteins, (ii) recruitment of endogenous N-WASP is unlikely, as this protein is strongly down-regulated at late stages of infection [10], and (iii) infection induces actin filament degradation, known to inhibit this transport system [30]. Interestingly, there is recent evidence of cross-talk between actin- and microtubule-dependent transport [23],[31],[32]. This raises the intriguing possibility that gelsolin-dependent actin processing might trigger the picking-up of virions or virus-loaded vesicles by the microtubule network for their transport from the nucleus to the periphery. This would be in agreement with the maintenance of microtubules until late in infection [10] and with the capsid-dynamin colocalization reported here.

Besides inducing the accumulation of gelsolin, MVM infection alters its subcellular distribution and membrane-binding affinity. This suggests that gelsolin may be subject to virus-induced post-translational modifications. This possibility is in keeping with the observation of multiple gelsolin species after SDS-PAGE. Although we were unable to immunoprecipitate enough endogenous gelsolin to allow characterization of its *in vivo* phosphorylation pattern, we present strong *in vitro* evidence of its regulation through phosphorylation by the NS1/CKIIα complex: NS1 can retarget CKII to gelsolin, leading the kinase to phosphorylate this protein at multiple sites. These modifications may be relevant to the role of gelsolin in virus egress, since CKII inhibition and gelsolin inactivation similarly impair the outward transfer of progeny virions from the nucleus.

Investigations in progress aim to pinpoint gelsolin functions involved in MVM infection, and particularly virus egress. As stated above, there may be a direct connection between gelsolin-dependent actin processing and virus transport systems. On the other hand, we show here that gelsolin also localizes to the cytoplasmic actin patches appearing late in infection and which are not associated with capsids. Although the role of these patches remains elusive, one might speculate that they fulfill a signaling function. Actin structures can indeed serve as scaffolds for signaling cascades, and because of its high affinity for PIP_2_, gelsolin is thought to affect cell pathways, notably ones involving PLC and PLD. Altogether, these observations point to lipid-dependent signaling as a potential alternative gelsolin target to be studied for its impact on the outcome of parvovirus infection.

## Materials and Methods

### Antibodies and reagents

#### Primary antibodies

Antibodies against actin (MP Biomedicals: C-4, 691002), gelsolin (BD Biosciences # 610413), α-tubulin, and myc-tag (Sigma: T6074; C3956), lamin B, and Lamp2 (Sta. Cruz Biotechnologies: M-20, sc 6217; C-20, sc8100), cathepsin B (Upstate Biotechnology: 06-480); neutralizing monoclonal anti-gelsolin antibody and control serum (Sigma: GS-2C4, 104K4781), rhodamine-coupled phalloidin (Invitrogen, R415). Rabbit antiserum recognizing the MVM NS1 protein (αNS1_C_) was raised with a peptide consisting of the 15 most C-terminal amino acids of NS1. Antiserum recognizing VP2 (αVP2) was raised with two peptides, MSDGTSQPDSGNAVH+C and SGNAVHSAARVERAA+C (Eurogentech). Monoclonal anti-capsid antibody B7 has been described [33].

#### Secondary IgGs

Horseradish-peroxidase-conjugated (HRP-conjugated) anti-rabbit and anti-mouse IgGs (Promega), HRP-conjugated anti-goat IgGs (Sta. Cruz), fluorescent-dye-labeled IgG (Dianova and Invitrogen).

#### Others

Glutathione Sepharose beads and columns, [^32^P]-labeled α-dCTP, γ-ATP (Pharmacia Amersham), [^32^P]-orthophosphate (MP Biomedicals).

### Cells and viruses

A9 mouse fibroblasts, derivatives thereof, and NCH149 human glioma cells [15] were maintained as monolayers in Dulbecco's Modified Eagle Medium (DMEM) containing 10% fetal calf serum (FCS). MVMp (MVM) and H1-PV were propagated respectively in adherent A9 and NCH149 cells. Virus stocks were prepared by freezing and thawing in TE pH 8.3. When indicated, full (DNA-containing) MVM particles were separated from empty capsids, on the basis of their buoyant density, by CsCl-gradient centrifugation.

### Plasmid constructs

#### Isolation of mouse gelsolin cDNA

A9 cDNA libraries were generated from mRNA preparations with the SMART^TM^ PCR cDNA synthesis kit (BD Biosciences). Full-length gelsolin cDNA was isolated in a single PCR reaction as described [3], using the N-terminal primer 5′-TGGTGGTGGAGCACCCCGAATTCCTGAAGGCAGGGAAGG-3′) and the C-terminal primer 5′-TCAGGCAGCCAGCTCAGCCAAGGCCCGGTCCAAAGGATCC-3 corresponding to the published mouse gelsolin sequence (NCBI NM 010354). PCR fragments were gel purified, cloned into pCR2.1 (Invitrogen), and sequenced (Microsynth GmbH, CH). This revealed the following differences with respect to the published amino-acid sequence: G36E, E213G, T214E, K245R, P274A, G374A, A566G, S599A, and N714D.

#### Production of the Y438A and D565N gelsolin mutants

Site-directed mutagenesis of gelsolin was performed by chimeric PCR [8] with an N-terminal primer (consisting of a unique Eco47III restriction site followed by the myc-tag sequence and then the first 40 nts of gelsolin) and a C-terminal reverse primer (consisting of a unique NotI site followed by 32 nts of coding sequence) together with two overlapping internal primers harboring the mutation (tyrosine 438 to alanine: 5′-CAG TTC GCT GGA GGC GAC AG-3′ and 5′-CT GTC GCC TCC AGC GAA CTG-3 or aspartic acid 565 to asparagine: 5′-G AAC TCC AAC AAC GCC TTT GTG-3′ and 5′-CAC AAA GGC GTT GTT GGA GTT C-3′). The Myc-tagged GlnY438A and GlnD565N mutants were subcloned into pCR2.1, sequenced, and transferred as Eco47III/Not1 restriction fragments into HpaI/NotI-cleaved pP38 [3], yielding pP38-MycGlnY438A and pP38-MycGlnD565N. pP38:GST-Tubα was made as described for pP38:GST-βactin [8] using the two overlapping internal primers (5′-ATCCTCCAAAATCGGATCTGATGCGTGAGTGCATCTCCAT-3′ and 5′-ATGGAGATGCACTCACGCATCAGATCCGATTTTGGAGGAT-3′) encompassing the fusion region. pEYFP-Tub (BD Biosciences) served as a template for α-tubulin sequences.

### Generation of stably transfected A9 cell lines

Stable transfectants were generated with pP38-MycGlnY438A, pP38-MycGlnD565N or pP38:GST-Tubα and the selection plasmid pSV2neo at the molar ratio of 25∶1 [3]. Colonies were pooled after growth under selection and frozen stocks prepared. Experiments were performed in absence of G418. Transfectants were kept in culture for less than 25 passages. Previously established cell lines contained pP38-GST-βactin, pP38-GST-TM5 [8], pP38-CKII:E81A [7], or pP38-PKCηT512A [3].

### MVM DNA replication in infected cells

Accumulation of MVM DNA was determined by Southern blotting [34]. When indicated, transfection with gelsolin-neutralizing antibodies was performed 4 h prior to virus infection, with 7 µg IgG and 15 µl Provectin (Imgenex). To prevent secondary rounds of infection, the cells were treated with 100 ng/ml neuraminidase (Sigma) 4 h p.i. They were harvested in TE buffer, digested with proteinase K, and total DNA was sheared by passage through a syringe. Viral DNA was analyzed by agarose gel electrophoresis and detected, after blotting onto nitrocellulose membranes, with a ^32^P-labeled probe corresponding to nts 385–1885 of the NS1-encoding region of MVM DNA.

### Production of infectious progeny viruses

To measure the formation and release of progeny virions, cultures were infected with MVM as described above. At the indicated times p.i., medium was removed and kept separately. Adherent cells were washed, harvested in DMEM without serum by scraping from the dish, and collected by centrifugation. Medium- and cell-associated virions were quantified, after repeated freezing and thawing, in standard plaque assays [5].

### Western blot analyses

Protein extracts were fractionated by discontinuous SDS-PAGE and blotted onto nitrocellulose membranes. Proteins of interest were detected by incubation for 18 h with appropriate primary antibodies in 10% dry milk/PBS and staining with HRP-conjugated secondary antibodies for 1 h followed by chemiluminescence detection (Amersham).

### Immunofluorescence microscopy

Cells were grown on spot slides (Roth), mock- or MVM-infected, and further incubated for the appropriate time. Cultures were fixed with 3% paraformaldehyde and permeabilized with 0.1% Triton X-100. Specimens were preadsorbed with 20% FCS, incubated with primary antibodies, and stained with specific Alexa Fluo 594-, CY2-, CY3-, or rhodamine-conjugated anti-species antibodies. After mounting with Elvanol, cells were analyzed by laser scanning microscopy with a Leica DMIRBE apparatus (63× lens, laser: red 543 nm, green 488 nm) and Powerscan software or by spinning disk confocal microscopy with a Perkin Elmer ERS 6Line microscope (100× lens, laser: red 568 nm, green 488) presenting a single slice of a stack. Quantitative analyses were performed on all slides of a stack, mean colocalization being calculated with ImageJ software.

### Biochemical fractionation of cell extracts

#### Association of proteins with cellular scaffolds and membrane structures [10]

Extracts were prepared and the insoluble material was separated from the soluble fraction by low-speed centrifugation. The pellet was extracted with 1% Triton X-100, after which the insoluble scaffold proteins (the iS fraction) were separated from the membrane-associated proteins by centrifugation (the supernatant was called the nM fraction). After low-speed centrifugation, the soluble components were further fractionated by high-speed centrifugation, yielding the cytosolic constituents (C) in the supernatant and a pellet that was extracted with Triton X-100, separating the soluble scaffold (sS-fraction) from the post-nuclear membrane fraction (pM). All volumes were adjusted to equivalent original cell numbers, making it possible to compare the relative amounts and distributions of selected proteins in differently treated cells.

#### Fractionation according to solubility [10]

Extracts were obtained by freezing and thawing (S1) and insoluble components were treated successively with equal amounts of CHAPS-buffer (S2), CHAPS-DOC-buffer (S3), and CHAPS-DOC-SDS-buffer (S4). The insoluble pellet (S5) was heated at 100°C in loading buffer. All fractions were analyzed by SDS-PAGE and western blotting.

#### Separation of nuclear, mitochondrial, and vesicular fractions from the soluble cytosol [6]

Nuclear components were obtained by pelleting at 900 g and further purification through 1 M sucrose. The 900-g supernatant was centrifuged at 2500 g to pellet large organelles like mitochondria in a “heavy mitochondrial fraction” (HMF), and the supernatant was centrifuged at 17000 g to pellet smaller organelles like vesicles in a “light mitochondrial fraction” (LMF). The final supernatant was considered to be the soluble cytosolic fraction. To determine association of newly synthesized virions with vesicles, the LMF suspended in hypotonic buffer was added to 50% iodixanol/142 mM sucrose (1∶2 v/v) and the components were separated according to their density by centrifugation for 4 h at 4°C in a self-forming gradient in a vertical rotor at 380,000 g. Fractions were collected from the top, volume-matched with the nuclear, HMF, and cytosolic fractions, and analyzed individually by Southern and western blotting.

### Isolation/quantitation of viral and cell components interacting with cytoskeletal proteins

Proteins and viral components interacting with cytoskeletal proteins were identified by affinity chromatography [8]. A9 cells or derivatives thereof (A9-P38:GST-βactin, A9-P38:GST-TM5, A9-P38:GST-Tubα) were infected (or not) with MVM (30 pfu/cell). Extracts were prepared as nuclear squeezes into the cytoplasm, loaded onto Glutathione Sepharose columns under isotonic conditions, and washed extensively. Components binding to the glutathione-S-transferase-tagged (GST-tagged) baits were then specifically eluted with 700 mM NaCl. Viral and cellular proteins were detected by western blotting. The single-stranded DNA genomes of infectious virions were detected by Southern blotting after extensive treatment with proteinase K.

### Immunoprecipitations

Protein extracts or fractions thereof were diluted in 700 µl Co-Ip-buffer (20 mM Hepes-KOH pH 7.5, 300 mM NaCl, 1 mM EDTA, 0.2 % NP-40) and pre-cleared by addition of FCS (5 µl) and protein G-Sepharose (40 µl) for 2 h at room temperature. After centrifugation, soluble proteins were incubated with specific antibodies/antiserum for 18 h at 4°C before addition of protein G-Sepharose for 2 h at room temperature. After extensive washes with Co-Ip buffer, immune complexes were collected and analyzed.

### In vitro kinase reactions

*In vitro* kinase reactions and tryptic phosphopeptide analyses were performed as described [35] with recombinant CKIIαβ (Roche) and the various PKC isoforms (Sigma). When indicated, purified GST-tagged wild-type or mutant (S473A) NS1 protein was added [8]. Gelsolin and tubulin used as substrates were produced in bacteria and purified as described for tropomyosin [8]. Assays were performed for 40 min at 37°C with 30 µCi γ-[^32^P]ATP in 50 µl of 20 mM HEPES-KOH [pH 7.5], 7 mM MgCl_2_, 150 mM NaCl, 1 mM DTT, in the presence of the appropriate cofactors. The reactions were stopped and the reaction products analyzed either directly or after immunoprecipitation, by 10% SDS-PAGE and semi-dry transfer onto polyvinyldifluoride (PVDF) membranes (Millipore). Phospholabeled proteins were then digested with trypsin and analyzed by two-dimensional thin-layer electrophoresis/chromatography (electrophoresis at pH 1.9/phosphochromatography).

## Supporting Information

Figure S1 (0.06 MB PDF)

Figure S2 (0.12 MB PDF)

Figure S3 (0.26 MB PDF)
