# Supplementary material for: Vesicular Egress of Non-Enveloped Lytic Parvoviruses Depends on Gelsolin Functioning
Source: PLoS Pathog. 2008 Aug 15;4(8):e1000126. doi: 10.1371/journal.ppat.1000126 (PMC2494870; doi:10.1371/journal.ppat.1000126)
Supplement: Figure S1 — (0.06 MB PDF) [file ppat.1000126.s001.pdf]

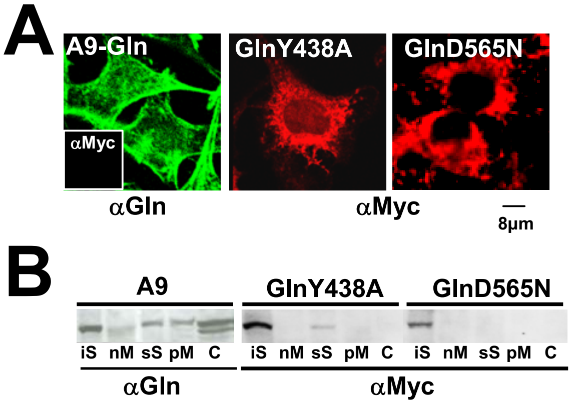

Supplement 1:
Asynchronously growing A9, or derivatives expressing dominant-negative gelsolin mutants (GlnY438A; GlnD565N) were infected with MVMp (30 pfu/cell) and analyzed 48 h p.i. (A) for the presence of recombinant gelsolin by immunofluorescence staining by means of their N-terminal myc-tag with αMyc. Endogenous gelsolin of parental A9 cells was shown for comparison using αGln. (B) For association with cellular scaffold and membrane structures by fractionating cell extracts using a combined sedimentation and Triton X-100 extraction procedure. The distribution of the respective proteins in the various fractions was determined by Western blotting using αMyc. Endogenous gelsolin of parental A9 cells was shown for comparison using αGln. iS, insoluble scaffold proteins; nM, mainly nuclear (membrane) constituents; sS, soluble scaffold proteins; pM, mainly vesicular and plasmic (membrane) constituents; C, cytosolic soluble proteins.
