# Supplementary material for: Vesicular Egress of Non-Enveloped Lytic Parvoviruses Depends on Gelsolin Functioning
Source: PLoS Pathog. 2008 Aug 15;4(8):e1000126. doi: 10.1371/journal.ppat.1000126 (PMC2494870; doi:10.1371/journal.ppat.1000126)
Supplement: Figure S2 — (0.12 MB PDF) [file ppat.1000126.s002.pdf]

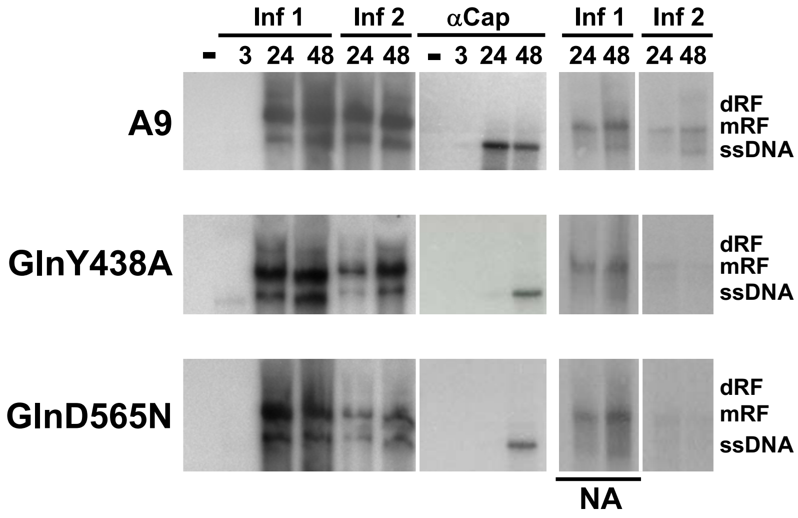

Supplement 2

Asynchronously growing A9, or derivatives expressing dominant-negative gelsolin mutants (GlnY438A; GlnD565N) were infected with MVMp (30 pfu/cell). When indicated cells were treated with neuraminidase (NA) after infection. Cells and supernatants were collected separately at indicated times p.i. (Inf 1). To measure infectious virions released into the medium, naïve cultures were inoculated with supernatants and harvested 24 h later (Inf 2). Alternatively virions shed into the medium were collected by immunoprecipitations with monoclonal anti-capsid antibodies B7 (αCap). Production of replicative intermediates (dRF, mRF) and progeny single-stranded virion DNA (ssDNA) were measured by southern blotting.
