# Supplementary material for: Vesicular Egress of Non-Enveloped Lytic Parvoviruses Depends on Gelsolin Functioning
Source: PLoS Pathog. 2008 Aug 15;4(8):e1000126. doi: 10.1371/journal.ppat.1000126 (PMC2494870; doi:10.1371/journal.ppat.1000126)
Supplement: Figure S3 — (0.26 MB PDF) [file ppat.1000126.s003.pdf]

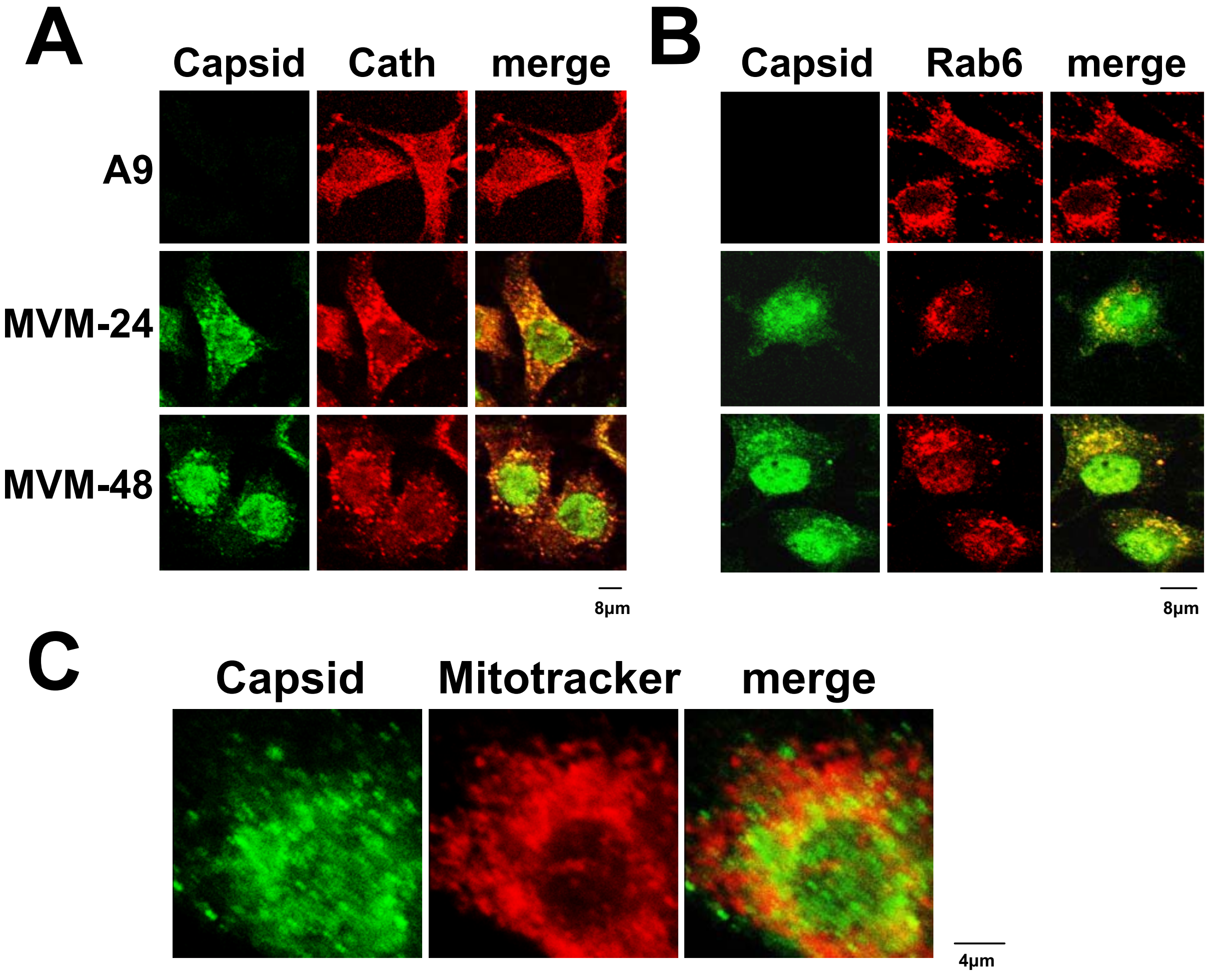

Supplement 3:
A9 cells and derivatives expressing GlnY438A or GlnD565N were grown on spot slides and infected with CsCl-gradient-purified MVM (30 pfu/cell) or mock-treated, and fixed with paraformaldehyde 24 and 48 h p.i. To avoid second-round infections, cells were treated with neuraminidase after infection. Capsids (detected with αB7 [green]) were analyzed by confocal laser scanning microscopy for their subcellular localization relatively to that of CathepsinB-positive (A) or Rab6-positive vesicles (B). Scale bar 8 µm. C, negative control, showing no colocalization with mitochodrial staining with mito-tracker. Colocalization regions appear as yellow in the merge
